# The DETAIL questionnaire is a useful and effective tool to assess spondyloarthritis in patients with inflammatory bowel disease

**DOI:** 10.3389/fmed.2023.1115362

**Published:** 2023-02-09

**Authors:** Onur Keskin, Bayram Farisogullari, Gozde Kubra Yardimci, Burcu Gurbuz, Melike Kole, Erkan Parlak, Omer Karadag, Taylan Kav, Umut Kalyoncu

**Affiliations:** ^1^Department of Gastroenterology, Hacettepe University School of Medicine, Ankara, Türkiye; ^2^Department of Rheumatology, Hacettepe University School of Medicine, Ankara, Türkiye; ^3^Department of Internal Medicine, Hacettepe University School of Medicine, Ankara, Türkiye

**Keywords:** IBD, DETAIL questionnaire, enteropathies, ulcerative colitis, Crohn’s disease

## Abstract

**Introduction:**

This study aimed to determine the effectiveness of adding a simple questionnaire related to musculoskeletal system to routine outpatient examination to detect undiagnosed axial and peripheral arthropathy in patients with inflammatory bowel disease (IBD).

**Materials and methods:**

A musculoskeletal symptom questionnaire was given to all patients with IBD during their follow-up examinations between January 2020 and November 2021. The DETAIL questionnaire consisting of six questions about the musculoskeletal system was administered by asking the patients with IBD. All patients who answered yes to at least one of these questions were directed to specialists in the rheumatology department to undergo a detailed examination. The patients who were diagnosed with rheumatological disease after further investigation were recorded. Patients with a known diagnosis of rheumatological disease were excluded from the study.

**Findings:**

There were 333 patients with IBD included in the study. Of these patients, 41 (12.3%) had a previously diagnosed rheumatological disease and were excluded from the evaluation. Of the remaining 292 patients (147 with ulcerative colitis, 139 with Crohn’s disease and six with indeterminate colitis; mean age 42 years), 67 (23%) answered yes to at least one of the questions and were referred to a rheumatology consultation. Rheumatological examination was completed in 52 patients. As a result of the evaluations, 24 patients (8.2%) were diagnosed with enteropathic arthritis (14 axial, 9 peripheral, and 1 axial plus peripheral). Patients with newly diagnosed enteropathy had a lower median disease age than patients without enteropathy.

**Conclusion:**

The DETAIL questionnaire is an effective and easy tool for identifying missed cases of SpA in patients with IBD.

## Introduction

Inflammatory bowel diseases (IBD) are life-long chronic diseases with an increasing incidence throughout the world. They comprise two main diseases, namely Crohn’s disease (CD) and ulcerative colitis (UC). Although the diseases have roots in the bowels, extraintestinal findings are frequently seen in both CD and UC. Almost half of patients with IBD experience an extraintestinal manifestation (EIM). IBD are systemic diseases that involve inflammatory pathology located outside of the bowels. Because of the shared inflammatory pathways, some EIM precede the IBD diagnosis. Common EIM include arthropathies (such as arthritis), metabolic bone diseases, eye diseases (uveitis and episcleritis), oral diseases (ulcerations), skin diseases (erythema nodosum, pyoderma gangrenosum), and hepatobiliary system involvement (1). Based on published results, the incidence of inflammatory arthropathy in patients with IBD is between 4 and 23% (2, 3). However, radiological studies conducted in this patient group also indicate the presence of subclinical or asymptomatic sacroiliitis (4).

Despite this high prevalence, diagnostic delay in inflammatory arthropathy is estimated to be around 8–11 years in patients with IBD. There are several possible reasons for this delay: The symptomatology and clinical spectrum of arthropathies may vary in patients with IBD, and underreporting of EIM by gastroenterologists because of a lack of awareness, time or diagnostic restraints. Spinal involvement and peripheral arthropathy may be seen in some patients with IBD, although the symptoms may not be severe and the clinical course may be silent. During outpatient visits with patients with IBD, gastroenterologists often do not ask about musculoskeletal symptoms unless the patient expresses a complaint. Some patients may have a limited knowledge of the possible relation between their bowel and joint complaints. Non-inflammatory joint complaints should be differentiated from inflammatory ones, especially in older patients with osteoarthritis or patients who perform manual labor. In contrast to non-inflammatory joint complaints, delays in the diagnosis of inflammatory arthropathy can lead to deterioration in quality of life and the development of chronic irreversible joint pathologies leading to loss of function in the long term (5, 6). While plain joint X-rays show the structural damage, these patients should be diagnosed at the very early stage of inflammation before irreversible joint destruction occurs. This early diagnosis could be possible with the proven, short, DETAIL Questionnaire directed to recognize signs and symptoms of inflammatory arthropathy (3).

In this study, we investigated the effectiveness of adding the DETAIL Questionnaire to routine patient examination to detect undiagnosed axial and peripheral arthropathy in patients with IBD. We administered the DETAIL Questionnaire to patients with IBD during their follow-up visits. Patients who answered yes to any of the questions underwent further examination.

## Materials and methods

### Patient selection

The study group comprised a cohort of patients with IBD with a confirmed diagnosis who had been followed up for more than 6 months at the Department of Gastroenterology. Patients with IBD who came to the outpatient between January 2020 and December 2021 were included in the study. IBD patients with severe disease findings and therefore hospitalized were not included in the study. Children younger than 16 years of age, pregnant women, individuals who did not want to participate in the study, and patients with IBD with severe systemic comorbidities were also excluded from the study.

There were 333 patients in the study who met these criteria. Of these patients, 172 were being followed up for CD, 155 for UC and six for indeterminate colitis. Among these patients, those with previously diagnosed rheumatological diseases were excluded from the study (41 patients, 23%). Of the excluded patients, 22 had axial and peripheral arthropathy, three had familial Mediterranean fever (FMF), two had psoriatic arthritis (PsA), two had Behcet’s disease (BD), two had BD + PsA, two had PsA + FMF, two had systemic sclerosis, one had FMF + ankylosing spondylitis, one had FMF + ankylosing spondylitis + BD, one had rheumatoid arthritis, one had systemic lupus erythematosus, one had juvenile rheumatoid arthritis + BD + FMF, and one had IgG4-related disease. The remaining 292 patients were questioned about the musculoskeletal system.

Approval was obtained from local ethics committee before starting the study.

### DETAIL questionnaire

The DETAIL questionnaire was used to enquire about the musculoskeletal system in the included patients (3). The attending gastroenterologist asked the following questions during the outpatient visits.

1. Have you ever had pain and swelling in your finger or toe joint and/or any joint for no other known reason?

2. Do your fingers or toes look swollen and sausage-like?

3. Do you have pain in your heels?

4. Have you ever had low back pain lasting at least 3 months without any trauma?

5. Do you have low back pain that improves with exercise in the morning and/or after rest?

6. Do you wake up at night because of back pain?

In case of positive (or yes) response to at least one of these questions, the patients were referred to the rheumatology department for further evaluation. It has been shown that post-test probability >75% can be obtained with at least three affirmative responses to DETAIL questionnaire. The aim of our study was to reach the diagnosis in patients whose musculoskeletal symptoms were not severe. Referring patients who answered positively to at least one question to the rheumatology consultation was an arbitrary decision we determined.

Patients who gave an affirmative response to at least one question on the DETAIL questionnaire were evaluated and examined by specialist rheumatologists in the rheumatology department one floor below in the same building.

### Rheumatological evaluation

Each patient was questioned in detail for Spondyloarthritis (SpA), and acute phase reactants and HLA B27 (if available) tests were performed. For peripheral joint involvement, possible arthritis [with the number of swollen joints (0–66 joints) and tender joints (0–68 joints)], enthesitis [with the Leeds (7), MASES (8) and SPRACC (9) enthesitis indexes] and dactylitis were first evaluated by physical examination. In addition, ultrasound (with gray scale and/or power Doppler imaging) was used to diagnose arthritis/enthesitis/dactylitis. In terms of axial involvement, each patient was first evaluated with sacroiliac X-ray and, if necessary, with sacroiliac magnetic resonance imaging (MRI). Patients diagnosed with enteropathic SpA by the clinician were classified as peripheral involvement (arthritis, dactylitis or enthesitis) or axial involvement (sacroiliitis and/or spinal involvement) considering the ASAS classification criteria (10).

### Statistics

Data were analyzed by using SPPS Statistics Version 21 (SPSS, Inc., IBM Corp., Armonk, NY, USA). Categorical variables are presented as the number of cases or percentages. Continuous variables are presented as mean ± standard deviation or median (minimum-maximum). The chi-square test was used to compare categorical variables. The non-parametric Mann–Whitney *U*-test was used to compare continuous variable. For all tests, *p* < 0.05 was considered to be statistically significant.

## Results

### Patients with IBD

The DETAIL questionnaire was administered to 292 patients, of whom 147 had UC (47.6% female), 139 had CD (48.2% female) and six had indeterminate colitis. The age (42.2 ± 15 years for UC vs. 42.1 ± 14 years for CD, *p* = 0.96) and disease duration [6 years (6 months-25 years) for UC vs. 4 years (6 months-40 years) for CD, *p* = 0.12] were similar in patients with UC and CD. Endoscopic findings of the relevant patients at the time of diagnosis were as follows: 44% of patients with UC had extensive colitis, 40% had left-sided colitis and 16% had proctitis. In patients with CD, 62% had small bowel involvement and ileitis, 28% had ileocolonic involvement and 10% had isolated colonic involvement. The clinical characteristics of the patients of the UC and CD patient groups are presented in Table 1.

**TABLE 1:** Clinical characteristics of UC and CD patients.

		Ulcerative colitis (*n*: 147)	Crohn’s disease (*n*: 139)	*P*-value
Age (mean ± SD)		42.2 ± 15	42.1 ± 14	0.96
Sex female *n* (%)		70 (47.6)	67 (48.2)	0.90
Disease duration (years) (median, min-max)		6 (0.5–25)	4 (0.5–40)	0.12
Disease location at diagnosis (%)	Proctitis	16		
	Left-sided	40		
	Colitis	44		
	Extensive		62	
	Colitis ileitis		28	
	Iliocolitis		10	
	Colitis			
Medications *n* (%)	Mesalazine	123 (83.6)	24 (17.2)	
	Azathioprine	47 (32)	101 (72.6)	
	Steroids	17 (11.5)	21 (15)	
	Biologics	26 (17.6)	58 (41.2)	
	No treatment	10 (6.8)	8 (5.7)	

The treatments are given in Table 1. Fistulising disease was present in 22 patients with CD (15.8%). Most of the patients in this group were receiving regular outpatient follow-up and did not have serious active gastrointestinal disease. For patients with UC, the current median Mayo score was 1 (0–6), while for patients with CD, the current median Harvey-Bradshaw Index was 2 (0–9).

### DETAIL questionnaire results

**FIGURE 1:**
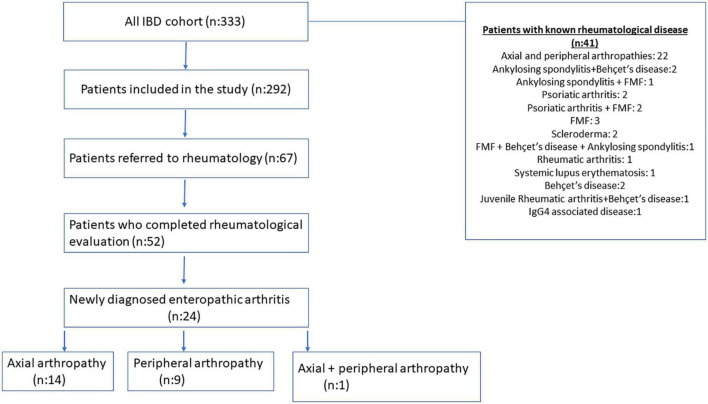
Flow charts for patients with inflammatory bowel disease (IBD).

Sixty-seven patients answered affirmatively to at least one question of the DETAIL Questionnaire (Figure 1). These patients were referred to the rheumatology department for a same-day examination. Fifteen patients did not go to the appointments for various reasons or did not complete the examination processes. The remaining 52 patients received a complete rheumatological examination, including blood and imaging tests. As a result of the detailed rheumatological examinations, 24 patients (8.2%) were diagnosed with enteropathic arthritis.

### Patients with enteropathic arthritis

When the clinical features of newly diagnosed patients with enteropathic arthritis were compared with other patients, the groups did not differ in terms of age, gender and disease type. The median disease duration was shorter in patients diagnosed with enteropathic arthritis than in other patients [4 years (6 months-18 years) vs. 6 years (6 months-18 years), *p* = 0.03] (Table 2).

**TABLE 2:** Clinical characteristics of patients with newly diagnosed enteropathy.

	Newly diagnosed enteropathy cases (*n*: 24)		No enteropathy (*n*: 268)		*P*-value
Age	46.2 ± 13.9		41.8 ± 15.0		0.16
Gender	13 F/11 M		127 F/141 M		0.33
Disease	11 CD	13 UC	128 CD	134 UC	0.83
	Ileitis (*n* = 8)	Proctitis (*n* = 1)	Ileitis (*n* = 78)	Proctitis (*n* = 23)	
	Ileocolitis (*n* = 2)	Left-sided colitis (*n* = 6)	Ileocolitis (*n* = 37)	Left-sided colitis (*n* = 52)	
	Colitis (*n* = 1)	Extensive colitis (*n* = 6)	Colitis (*n* = 13)	Extensive colitis (*n* = 59)	
Median disease duration	4 years (6 month-18 years)		6 years (6 month-18 years)		0.03

Of the patients with enteropathic arthritis, 14 had only axial involvement. Sacroiliitis was demonstrated by sacroiliac MRI in 11 patients and by sacroiliac X-ray in three patients. In addition, peripheral involvement—namely arthritis, which was detected by physical examination and confirmed by ultrasound—was present in nine patients. Of these patients, three had polyarthritis, three had oligoarthritis, and three had monoarthritis. In terms of the joint localizations, five had arthritis in the knee, four in the wrist, two in the ankle, two in the hand MCP/PIP and in one in the sternoclavicular joint. One patient, who had CD, was classified as axial plus peripheral involvement due to MRI-confirmed sacroiliitis and ultrasound-confirmed hip and knee synovitis. HLA B27 was positive in 2/20 patients (10%; one with axial and one with peripheral involvement) and negative in 18/20 patients (90%). In patients with axial involvement, 8/14 (57%) had UC and 6/14 (43%) had CD. Similarly, in patients with peripheral involvement, 5/9 (56%) had UC and 4/9 (44%) had CD.

## Discussion

In this study, application of the simple DETAIL Questionnaire resulted in a new rheumatological disease diagnosis in 8.2% of patients being followed up for IBD and without a previous diagnosis of rheumatological disease. These patients generally have mild symptoms and do not express musculoskeletal complaints unless asked. Hence, the disease is missed for a long time, and most patients are diagnosed in the advanced stages of the disease.

Our patient group consisted of 333 patients, of whom 41 patients (12.3%) had a known rheumatologic disease. In the remaining 292 patients, after administering the DETAIL Questionnaire, 24 patients received a rheumatologic disease diagnosis as a result of rheumatology consultation. Thus, the rate of rheumatologic disease as an extraintestinal finding in our IBD cohort increased to 19.5%.

In addition to the gastrointestinal tract, IBD can affect many other organs. This phenomenon impairs the quality of life of patients with IBD and even worsens quality of life in some patients more than intestinal disease. The reported frequency of EIM is quite variable—between 6 and 47% (11)—due to heterogeneity among the studies and the changes in the definition of the diseases. In a large Swedish study, the frequency of EIM was 29% in patients with IBD. In addition, more than one EIM was seen in approximately 37% of these patients (12). Although the pathogenesis of EIM development is not fully known, it is thought that enteric bacteria leaking from the diseased and permeable intestinal mucosa and host major histocompatibility complexes in other organs have shared epitopes, and the adaptive immune system response triggered and plays a role in the pathogenesis (11, 13). Autoimmune response exacerbated by genetic predisposition is also thought to play a role in the pathogenesis of the disease (14). Patients with EIM often require a multidisciplinary treatment approach—thus, they are not followed up by gastroenterologists alone. Although there is no clear recommendation regarding the screening of IBD patients for SpA in the guidelines, it is recommended that individuals under the age of 40 with inflammatory low back pain be screened with imaging methods (1).

Diagnostic delay is frequent, mostly in HLA B27-negative spondyloarthropathies, and this phenomenon is not acceptable, especially when pathology is accompanied by other inflammatory diseases (15). In clinical practice, it would not be practical to refer every patient with IBD to a dermatologist, rheumatologist or ophthalmologist. Targeted surveys like the DETAIL Questionnaire should be created for other EIM. They could be easily implemented in clinical practice—because they take little time to apply—and would ensure that suspicious cases are identified and referred to the relevant departments for consultation. The Toronto Axial Spondyloarthritis Questionnaire, which was created for this purpose in patients with IBD, queries about the presence of back pain and stiffness for the last 3 months, and further detailed inquiries are made to patients who gives positive answers (16). In addition, Queira et al. applied a questionnaire consisting of three questions to evaluate axial and peripheral arthritis in IBD patients, and 27 and 32% of the patients, respectively, answered two of the three questions affirmatively, and the patients were considered positive. Sensitivity and specificity of both the axial and peripheral questionnaire was >80% (17). It is difficult to state that these scorings have sufficient validations in terms of effectiveness. The DETAIL Questionnaire, which was developed later, is a more easily applied test consisting of six questions asking about axial and peripheral arthropathy. The validation study of the DETAIL questionnaire was conducted in a multicentre study and published (18). In our patient group, peripheral arthropathy was found at an almost similar rate to axial arthropathy in newly diagnosed enteropathic arthritis cases, which is consistent with the literature (3, 19). Specifically, 13 patients were diagnosed with axial arthropathy and 10 patients were diagnosed with peripheral arthropathy. Among those diagnosed with axial arthropathy, five had CD and eight had UC. In those diagnosed with peripheral arthropathy, five had CD and five had UC.

It is possible to ask all six questions in about a minute during the routine clinical history taking. Although we did not measure the administration time in this study, it was reported to be as quick as 0.8 ± 0.3 min in the original article. As the previous studies support, we believe the DETAIL Questionnaire is easy to use and not time-consuming. It is clear that the DETAIL questionnaire can be easily used in routine IBD outpatient follow-up. It is clear that a questionnaire that will reveal critical questions to other EIMs, especially to identify common EIMs, can provide significant improvement in patient follow-up.

A strength of our study is that we conducted it in a well-defined and followed up group of patients with IBD. Another strength is the successful collaboration we have established with the rheumatology department in the same institution. Most patients received a same-day evaluation by expert rheumatologists. A limitation of our study is that the DETAIL Questionnaire was administered by more than one gastroenterologist, although the same questions were asked in full.

In the long-term follow-up of patients with IBD, focusing only on the gastrointestinal symptoms and signs may lead to missing other potentially important pathologies, including EIM. Perhaps the most common and one of the most important of these is spondyloarthropathies. Referral of all patients with IBD to the rheumatology department is neither possible nor feasible in current practice. However, directing suspicious cases to a rheumatologist based on screening with a simple and quick questionnaire by the gastroenterologist would make the early diagnosis of axial spondyloarthritis and peripheral arthropathies possible. There is no doubt that early use of appropriate treatments may increase the quality of life of the patients and prevent the development of morbidities of these pathologies. The issue of how the treatment should be applied after the detection of rheumatological patients in IBD patients is also requires the collaboration of gastroenterology and rheumatology departments. In our patient group, after the diagnosis of rheumatological disease, sulfasalazine was added to the treatment in some patients, and biologic agents were required in some patients followed up with conventional treatment.

## Conclusion

We used the easily applicable DETAIL Questionnaire in our patients and detected EIM involving the musculoskeletal system in approximately 8% of our patient cohort. We think that the DETAIL Questionnaire is a useful questionnaire that can be easily applied in IBD outpatient clinics.

## Data availability statement

The raw data supporting the conclusions of this article will be made available by the authors, without undue reservation.

## Ethics statement

The studies involving human participants were reviewed and approved by the Hacettepe University Local Ethics Committee. The patients/participants provided their written informed consent to participate in this study.

## Author contributions

OnK, BG, MK, EP, OmK, and TK involved in acquisition and in the analysis and interpretation of data and wrote the first draft of the manuscript. BF wrote the first draft of the manuscript. GY involved in acquisition and in the analysis and interpretation of data. UK designed the study, involved in acquisition and in the analysis and interpretation of data, supervised the study related procedures, and critical revision of the manuscript. All authors contributed to the article and approved the submitted version.
